# Compact and ultra-efficient broadband plasmonic terahertz field detector

**DOI:** 10.1038/s41467-019-13490-x

**Published:** 2019-12-05

**Authors:** Yannick Salamin, Ileana-Cristina Benea-Chelmus, Yuriy Fedoryshyn, Wolfgang Heni, Delwin L. Elder, Larry R. Dalton, Jérôme Faist, Juerg Leuthold

**Affiliations:** 1 ETH Zurich, Institute of Electromagnetic Fields (IEF), 8092 Zurich, Switzerland; 2 ETH Zurich, Institute for Quantum Electronics (IQE), 8093 Zurich, Switzerland; 3 Department of Chemistry, University of Washington, Seattle, WA 98195-1700 USA; 4 Present Address: Harvard John A. Paulson School of Engineering and Applied Sciences, Harvard University, Cambridge, MA 02138 USA

**Keywords:** Nanophotonics and plasmonics, Integrated optics, Silicon photonics, Nonlinear optics, Terahertz optics

## Abstract

Terahertz sources and detectors have enabled numerous new applications from medical to communications. Yet, most efficient terahertz detection schemes rely on complex free-space optics and typically require high-power lasers as local oscillators. Here, we demonstrate a fiber-coupled, monolithic plasmonic terahertz field detector on a silicon-photonics platform featuring a detection bandwidth of 2.5 THz with a 65 dB dynamical range. The terahertz wave is measured through its nonlinear mixing with an optical probe pulse with an average power of only 63 nW. The high efficiency of the scheme relies on the extreme confinement of the terahertz field to a small volume of 10^−8^(*λ*_THz_/2)^3^. Additionally, on-chip guided plasmonic probe beams sample the terahertz signal efficiently in this volume. The approach results in an extremely short interaction length of only 5 μm, which eliminates the need for phase matching and shows the highest conversion efficiency per unit length up to date.

## Abstract (web summary)

Most efficient terahertz detection schemes rely on complex free space optics and require high-power lasers. Here, the authors report an integrated plasmonic terahertz detector on a silicon photonics platform, with 2.5 THz bandwidth and a 65 dB dynamical range operating at an optical power of only 63 nW.

## Introduction

Terahertz (THz)^1,2^ research is gaining a new momentum thanks to recent breakthroughs in the areas of quantum electrodynamics^3–5^, ultrafast nanoscopy^6^, spectroscopy^7,8^, telecommunications^9^, and imaging^10^. Yet, a real outbreak of THz technology has been hindered so far by the impracticality of nowadays THz systems that require bulky free-space optics. In addition, the nonlinear mixing with the probe pulse typically occurs in exotic material systems, such as e.g. zinc-telluride (ZnTe)^11^ or gallium arsenide (GaAs)^12^, and a high sensitivity is only reached at the expense of a high laser power^13^. Chip-scale, high-performance and broadband THz systems that exploit well-established silicon-on-insulator (SOI) technology and operating at low optical powers could pave the way to a multitude of new applications^14^.

Most widely used room-temperature THz detection approaches rely on photoconduction^15,16^ or nonlinear parametric up-conversion in a crystal with *χ*^(2)^ second-order susceptibility^17^. In both cases, the electric field of a THz wave is measured indirectly, by means of its modulation onto an optical probe^1^. Photoconductive antennas (PCA) produced on low-temperature grown GaAs (LT-GaAs)^12^ or indium gallium arsenide (InGaAs)^18^ substrates are relatively compact and have already demonstrated high sensitivities when integrated with plasmonic electrodes^19^. While these detectors feature a broad bandwidth and a very high dynamical range at short measurement acquisition times, a fine balance has to be found between the carrier mobility and lifetime to ensure an efficient yet broadband detection^20^. This compromise typically results in an efficiency that drops beyond 1 THz. Conversely, detectors relying on the *χ*^(2)^ Pockels effect should offer higher speed. For instance, crystals such as lithium niobate (LiNbO_3_) or ZnTe have been used to detect THz and mid-IR fields^11,21,22^. Unfortunately, the *χ*^(2)^ effect is rather weak and comes with a small photon-to-photon up-conversion efficiency, which requires large interaction lengths and in return needs phase matching of the interacting waves^23^. Nonetheless, impressive results have been demonstrated with field sensitivities equivalent to a single photon^3^ or vacuum field fluctuations^4^. As an alternative to inorganic crystals, nonlinear organic (NLO) material systems demonstrated record high *χ*^(2)^ nonlinearities^22,24^. They already have demonstrated THz detection up to 15 THz^25^. Still, electro-optic detection is quite cumbersome as it relies on bulky free-space optics and rather large nonlinear crystals. Additionally, a compromise needs to be found between an ideally focused beam and a long interaction length in order to maximize the conversion efficiency. In a first approach^26^ to miniaturize these detectors, an antenna was used to collect and confine the THz field to a small volume of 44 μm^3^ where an electro-optic polymer was located. An electro-optic bandwidth of 1.25 THz was reported with a dynamical power range of ~40 dB. In this way, phase-matching between the THz wave and optical signal was avoided. Yet, the efficiency was limited by the weak optical probe confinement of the free-space setup. This led to an inefficient nonlinear interaction with the THz signal and accordingly to a low efficiency. Recently, integrated plasmonic waveguides^27^ functionalized with nonlinear materials have shown impressive nonlinear *χ*^(2)^ efficiencies^28^. Meanwhile, they have demonstrated good performance as electro-optical modulators for high-bit rate systems up to 120 GBd^29^ or for the direct electro-optical up-conversion of 60 GHz microwave signals^30,31^. More recently, the frequency response of these plasmonic modulators has been shown to exceed 500 GHz^32^. The question that we address here is the following one: Can the combination of plasmonics with an organic nonlinear material offer a simple path towards integrated, efficient broadband THz detectors?

To answer this question, we introduce an ultra-compact-integrated plasmonic THz field detector realized on a silicon platform. The detector directly measures the electric field amplitude and phase of a THz signal in absolute units (V/m). Despite the small size of the detector, we show one of the highest conversion efficiencies which, per unit length, is 1000–10,000 times higher than in bulk nonlinear crystals. The high conversion efficiency is related to the strong confinement of the THz wave and optical probe signal in the nonlinear plasmonic waveguide and a multi-resonant antenna design. Here, multi-resonant means that different spectral ranges are covered by individual resonant antennas to provide an overall broadband response. The detector is used to perform THz time-domain electro-optic sampling. As a result, we demonstrate an electro-optic bandwidth of 2.5 THz with a dynamical range of 65 dB at an optical probe power of 63 nW. Also, we provide a method to retrieve the amplitude of an arbitrary input THz pulse. Finally, we compare the proposed approach with established THz time-domain spectroscopy (TDS) techniques and discuss the advantages of our implementation.

## Results

### On-chip THz detection

**Fig. 1:**
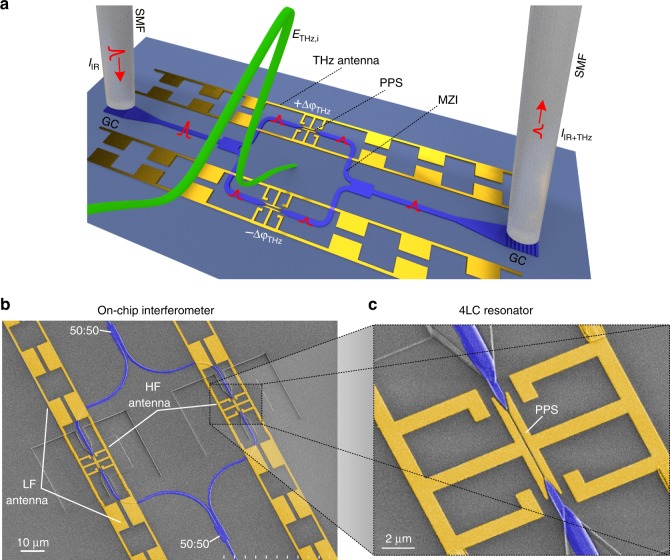
On-chip terahertz detector. **a** The detector consists of a Mach–Zehnder interferometer (MZI) with antenna-coupled plasmonic phase shifters (PPS). An incident THz field (*E*_THz,i_) introduces, via the linear electro-optic effect, a refractive index change in the gap of the two antenna-coupled phase shifters. This change of refractive index leads to a linear phase delay (±Δ*φ*_THz_) of the IR probe pulses (*I*_IR_) traveling through the phase shifters. The phase modulation experienced by the IR probe pulses is transformed into an intensity modulation (*I*_IR+THz_) at the output of the interferometer. The probe pulses are coupled in and out on-chip silicon (Si) waveguides by means of grating couplers (GC). The organic electro-optic material in the two-phase shifters located in opposite arms of the MZI has opposite polarity to enable push-pull operation. Scale bar is 10 μm. **b** False color scanning electron image of the fabricated multi-resonant THz detector. The antenna comprises of a high-frequency (HF) antenna and a low-frequency (LF) antenna. Each antenna is resonant within a spectral range. Combined they provide a broadband THz response. **c** Close-up view of the HF-THz antenna directly coupled to the PPS. Scale bar is 2 μm. SMF single mode fiber, 50:50: multi-mode interferometer.

A schematic view of the on-chip THz detector and its operation principle is given in Fig. 1a. The detector comprises a photonic Mach–Zehnder interferometer (MZI) in silicon (Si) with THz plasmonic phase-shifters (PPS). The PPSs consist of a THz antenna with two metallic arms forming a plasmonic slot waveguide filled with a nonlinear *χ*^(2)^ material^33^. An incident THz field (*E*_THz,i_) excites free charges in the metallic antenna (shown in gold color), which accumulate across the antenna gap^30^. The accumulated charges produce an electric field *E*_THz,g_ across the gap, which changes the refractive index in the slot waveguide by means of the linear electro-optic effect, i.e. the Pockels effect^34^. An optical probe (*I*_IR_) launched into the two branches of the MZI is converted into surface plasmon polaritons (SPPs) at the plasmonic phase shifters where they experience the refractive index changes. Finally, the SPPs are converted back to Si waveguides and interfere at the output of the MZI. The intensity at the output of the MZI (*I*_IR+THz_) follows a cosine-shaped transfer function^34^ that depends on the phase delay (±Δ*φ*_THz_) induced by the refractive index change. The MZI is adjusted to operate in the quadrature point, where the output intensity is linearly related to the incident THz signal amplitude. The output optical intensity *I*_IR+THz_ is thus given by

1 $$I_{{\mathrm{IR}} + {\mathrm{THz}}} = \frac{{I_{{\mathrm{in}}}}}{2}\left[ {1 + {\mathrm{cos}}\left( {\frac{{\pi }}{2} + 2\,{\mathrm{\Delta }}\varphi _{{\mathrm{THz}}}} \right)} \right] \approx \frac{{I_{{\mathrm{in}}}}}{2}(1 + 2\,{\mathrm{\Delta }}n_{{\mathrm{eff}}}k_0L_{{\mathrm{slot}}})$$

with *k*_0_ = 2*π*/*λ*_0_ the wavenumber, *λ*_0_ = 1560 nm the probe wavelength, *L*_slot_ = 5 μm the length of the plasmonic slot. Δ*n*_eff_ is the change of the effective group index of the probe pulse in each PPS^35^, which changes linearly with the THz field *E*_THz,g_ (see the “Methods” section and Supplementary Note 4). A detailed analysis of the detector’s performance with respect to the MZI operation point is discussed in Supplementary Notes 2 and 3.

The THz antenna consists of two parts, a high-frequency (HF) and a low-frequency (LF) resonant antenna (see Fig. 1b). The combination of the two resonators provides a broadband frequency response. The HF THz resonator antenna (>1 THz) contains a four-leaf clover (4LC) structure with the antenna gap forming the plasmonic waveguide. The LF resonators are formed by photonic-bandgap (PBG) lines designed to enhance the field at lower frequencies (<1 THz) without affecting the HF resonance of the 4LC structure. The exact dimensions of the capacitive and inductive elements of the antenna provide a way to tailor the frequency response of the detector by its geometry (see Supplementary Note 1).

A strong THz-to-optical up-conversion efficiency can be expected for multiple reasons in this design. First, the THz wave collected by the antenna induces a voltage across the highly sub-wavelength gap and results in a very strong field. Further, the field is enhanced by the multi-resonant antenna design. These two aspects alone provide an enhancement of the THz field in the order of ~1000. Then, the active material in the slot is highly nonlinear (*r*_33_ ≈ 120 pm V^−1^). Finally, the plasmonic architecture provides an almost perfect overlap of the probe pulse and the THz wave with the nonlinear material in a slow-light-enhanced plasmonic gap waveguide (see Supplementary Note 4). The combination of these characteristics allows a very short active section length, where walk-off between the two signals is avoided and the need for phase matching is eliminated. In addition, the short plasmonic section keeps the optical losses to a miminum.

The devices have been realized on a Si wafer using an in-house SOI technology. Details on the fabrication are given in the “Methods” section. A close-up view with scanning electron microscope images of the fabricated MZI and THz antenna is shown in Fig. 1b, c. Two THz detectors have been fabricated with 4LC HF resonances around 1.2 and 2.4 THz, respectively.

### Time-domain electro-optic sampling experiment

**Fig. 2:**
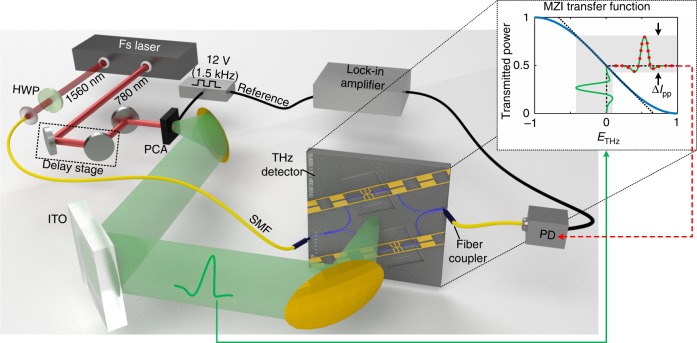
THz time-domain electro-optic sampling setup. A dual wavelength laser system provides the pump pulses (780 nm) and the optical IR probe signal (1560 nm). The 780 nm signal illuminates the PCA and drives the generation of phase-locked THz pulses. A square bias with a frequency of 1.5 kHz and an amplitude of 12 V is applied to the PCA. The IR probe beam is coupled by means of a single mode fiber (SMF) directly to the on-chip Si input waveguide. A half-wave plate (HWP) controls the linear polarization state of the IR probe to be oriented perpendicular to the plasmonic gap. A motorized delay stage controls the relative delay between the THz signal and IR probe. The probe signal is then guided through the MZI configuration where it probes the amplitude of the THz signal. The output intensity of the probe signal depends on the THz signal as dictated by the MZI transfer function (see inset). The red points depict different relative delays between the optical and THz pulses. Finally, a lock-in amplifier is employed to demodulate the electro-optic signal at the frequency of the driving voltage of the PCA.

The performance of the THz field detector is assessed in an electro-optic sampling experiment, where the complex response of our detector (amplitude and phase) is determined in the time-domain. The measurement setup is depicted in Fig. 2. A femtosecond (fs) laser provides fundamental pulses (~150 fs) at an infrared (IR) wavelength of 1560 nm and phase-locked frequency-doubled pulses (780 nm). The latter pulses are used to generate chopped THz pulses by means of a PCA. The THz pulses are focused by a 90° off-axis parabolic mirror onto the chip. We use as a probe signal the fundamental pulses. These IR pulses are coupled into a single mode fiber (SMF) and directly fed to the Si input waveguide of the THz detector. At the output, the intensity of the probe signal is measured by a photodiode. The modulation induced by the chopped THz signal is detected with a lock-in amplifier as a function of the relative delay between the IR and THz pulses. A motorized delay stage controls the relative delay between both pulses.

**Fig. 3:**
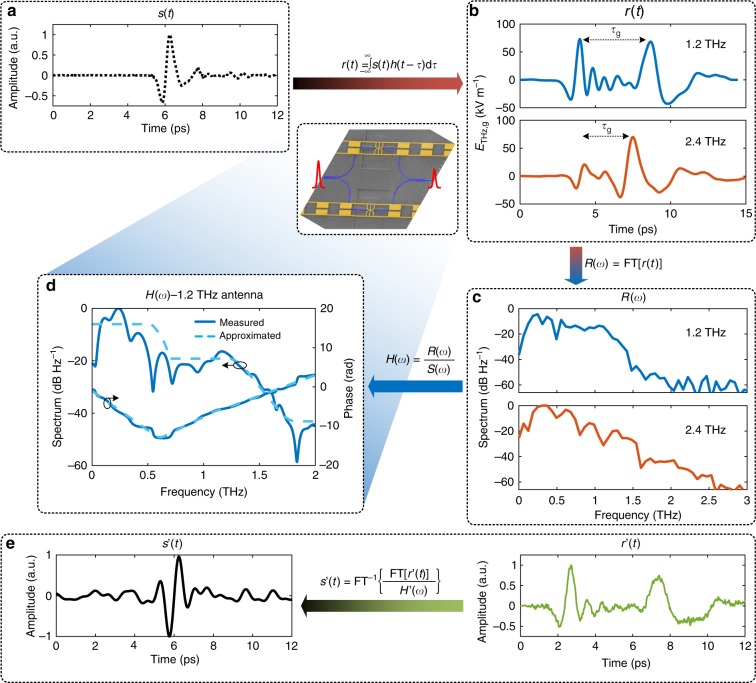
Experimental results. **a** Time domain signal of the PCA source measured with a 200 μm ZnTe crystal. **b** Electric field amplitude of the THz pulse in time domain detected by the MZI detectors with center frequencies of 1.2 and 2.4 THz, respectively. In both cases, the initial pulse of the detected time trace is attributed to the response of the HF-THz antenna component. The following slow THz pulse originates from the response of the LF antenna component. The detected signal is the convolution of the source *s*(*t*) with the impulse response of the detector *h*(*t*). **c** The Fourier transform (FT) of the THz time traces for the 1.2 and 2.4 THz detector, respectively. In both cases, the dynamic range is ≥60 dB for an integration time of 20 s per point with an optical probe power of 63 nW. **d** Measured (solid blue lines) and approximated (dashed light blue lines) complex frequency response of the detector. The frequency response of the detector can be computed from the measured spectrum in **c** and the spectrum of the known source in **a**. **e** Example of a retrieved input signal (black curve) as derived from a measured time trace (green curve) using the approximated frequency response in **d**.

Figure 3 presents the main results of the electro-optic-sampling experiment where the experimental complex transfer function of the detector (Fig. 3d) is measured. Furthermore, we show that this knowledge can be used to retrieve the time-domain profile of any arbitrary input signal (Fig. 3e). We start the discussion with Fig. 3a, which shows the temporal evolution of an input THz field (*E*_THz,i_) as detected by a ZnTe crystal of 200 μm length (see the “Methods” section and Supplementary Note 6). The measured impulse response (*E*_THz,g_) obtained with the 1.2 and 2.4 THz detectors are depicted in Fig. 3b. A clear low-noise waveform is found for the two detectors. Each of the two responses exhibit two distinct pulses spread in time by *τ*_g_. The first pulse is mainly the impulse response of the HF antenna part optimized for the reception of signals around 1.2 and 2.4 THz, respectively. This pulse is followed by a broader pulse which comes from the LF antenna response. The spread in time-domain is caused by the dispersive nature of the THz antenna. The 2.4 THz detector’s initial pulse has a lower amplitude due to a lower field amplitude of the source at higher frequencies and due to a reduced field enhancement at these frequencies.

The Fourier transform of the recorded time traces *R*(*ω*) are reported in Fig. 3c. They reveal the frequency responses of the measured signals. Both detectors exhibit an electro-optic bandwidth exceeding 2 THz with respective dynamic ranges of 60 and 65 dB (in field amplitude squared *|E*_THz,g_|^2^) measured with an optical probe power of 63 nW. The 1.2 THz detector features a good signal-to-noise ratio up to 1.2 THz. The 2.4 THz detector drops off beyond 600 GHz. Yet, it provides a much better power spectral density between 1.5 and 2.5 THz.

We derive the detector’s complex transfer function *H*(*ω*), with the purpose to provide a method to remove the dispersion introduced by the dual-antenna. We start from the measured output *R*(*ω*) and input *S*(*ω*) frequency responses and find

2 $${H}\left( {\it{\omega }} \right) = \frac{{{R}\left( {\it{\omega }} \right)}}{{{S}\left( {\it{\omega }} \right)}}.$$

The complex-valued frequency response of the 1.2 THz detector is plotted in Fig. 3d (solid blue lines). The magnitude of the frequency response $\left| {H\left( \omega \right)} \right|$ reveals two distinct frequency domains (see Fig. 3d) (left *y*-axis). A first region extends up to about 0.65 THz and obviously shows the response of the LF part. A second range with a weaker response extends up to about 1.2 THz. This response can be associated to the 4LC resonator. Next, we investigate the phase response *φ*_H_ = arg[H(*ω*)] (see Fig. 3d) (right *y*-axis). The phase is linear and has two distinct characteristic slopes in the two frequency domains. This results from the chirp experienced by the detected pulse, which we describe by the group delay

3 $$\tau _{\mathrm{g}} = - \frac{{{\mathrm{{d}}}\varphi _{\mathrm{H}}}}{{{\mathrm{{d}}}\omega }}.$$

The phase-slopes in Fig. 3d correspond to two distinct group delays that are offset by 5.25 ps. They are attributed to the impulse response delays of the two antenna parts. We further approximate the measured magnitude frequency response by a step filter combined with an exponential roll-off, see dashed light blue lines in Fig. 3d. The first step response stems from the LF filter which sets in with a group delay of 5.25 ps. The second step response can be associated to the 1.2 THz 4LC antenna frequency response. Likewise, we approximate the phase response of the detector by constant group delays, corresponding to a linear phase relation (dashed light blue lines). The two slopes of the phase response correspond to a constant group delay offset of the said 5.25 ps.

The knowledge of the complex-valued frequency response can now be used to retrieve the time-domain profile of an arbitrary input THz signal from the detected one. When e.g. applying the idealized frequency response *H*′(*ω*) to a sampled signal from a different measurement *r*′(*t*) obtained with a similar detector, we can derive the input signal *s*′(*t*) (see Fig. 3e). A comparison with the input signal as measured with the ZnTe crystal shows a strong similarity. This shows how the approximated transfer function would allow to retrieve an unknown THz pulse fairly well. The same is valid for the 2.4 THz detector (see Supplementary Note 5). It should be noted that for applications where a strong group delay should be avoided, a simpler antenna (e.g. bow-tie antenna) can be used to minimize the influence of the antenna on the measured THz pulse.

## Discussion

**Fig. 4:**
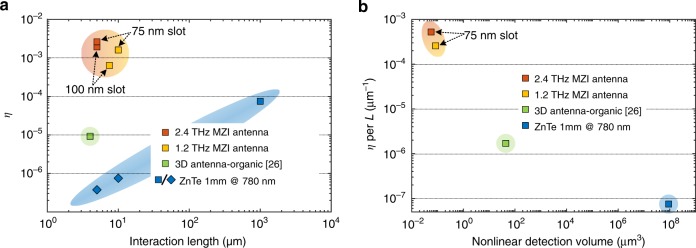
On-chip detector performance. **a** The modulation efficiency as a function of the nonlinear interaction length is presented for several devices (with a slot width of the PPS of 75 or 100 nm, respectively). For comparison, we show the modulation efficiency of the 3D antenna and a ZnTe crystal of 1 mm length. The rhombus shapes represent extrapolated values from the detection efficiency at 1 mm length. **b** The modulation efficiency per unit length of interaction distance is represented as a function of the nonlinear detection volume in the different platforms.

In Fig. 4, we compare the efficiency of our detectors with different platforms, namely a 1 mm long bulk ZnTe crystal as a lab standard and the 3D polymer-antenna^26^ mentioned before. For this purpose, we analyze the total peak-to-peak modulation Δ*I*_pp_ of the probe intensity (see inset Fig. 2) normalized by the detected average intensity *I*. We name this ratio the modulation efficiency *η*, i.e. *η* = Δ*I*_pp_/*I*. From Fig. 4a one can see that the 2.4 THz detector (red square) featuring only 5 μm interaction length is 35 times more efficient than a 1 mm long bulk ZnTe crystal (blue square). To shed some light on the high efficiency, we plot in Fig. 4b the modulation efficiency per unit length as a function of the nonlinear detection volume. The efficiency per unit length increases dramatically when transitioning from detection in free space (blue square) to detection in a confined volume (nonlinear interaction volume) of a few cubic micrometres (green square) and then, as in this case, to below 1 μm cube. In fact, in this configuration the THz field has been confined in a compact volume of ~0.05 μm^3^, which corresponds to a sub-wavelength volume of 10^−8^(*λ*_THz_/2)^3^. A detailed comparison between different technologies for THz electric field measurement is presented in Supplementary Note 7.

New opportunities will emerge from the unique advantages offered by integrated detection. For instance, more advanced detection schemes become possible with the guided optical probe by taking advantage of parallelization. A detector array sampling the same focused beam could reduce the size mismatch between the detector and the focused THz beam spot, including beam steering. In addition, more complex detection schemes are possible to measure correlation functions^3^. Finally, the advantage of low optical powers means a fully integrated THz system on silicon^36^ with lasers^37–39^, frequency combs^40^ and photodetection^41^ is possible.

In conclusion, we have demonstrated a fully monolithic THz field detector that can sample broadband THz pulses with record-high efficiency. The compact and integrated architecture is combined with a single photodiode that is fiber-coupled directly from the chip. This performant yet handy design might enable new spectroscopic applications, open new avenues for cavity quantum electro-dynamics in the time-domain^4,42^ or trigger new venues for THz communications^43^. Finally, the demonstrated platform could pave the way towards a new generation of hand-held THz systems operating at lowest power.

## Methods

### Device fabrication

Devices were fabricated in-house on a SOI wafer. First the silicon access waveguides were fabricated by e-beam lithography and dry etching. An alumina etch stop layer (100 nm) is then deposited. Subsequently, a silicon dioxide (700 nm)-cladding layer was deposited on top of the silicon waveguide and locally etched at the device locations (see Supplementary Note 2). The plasmonic phase shifters and THz antennas were formed by e-beam evaporation of Ti/Au (2 nm/150 nm) metal layers and a lift-off process. Finally, the organic electro-optic material composite (3:1 HD-BB-OH:YLD124) was applied by spin-coating on the chip and baked. Electric-field poling to induce opposite alignment (push-pull configuration) in the two branches of the MZI was carried out by standard methods^34^. A macroscopic nonlinear coefficient around *r*_33_ ≈ 120 pm V^−1^ was characterized. Two gap widths are investigated, i.e. 75 and 100 nm. The total optical losses of the integrated plasmonic detector amount to ~31 dB.

### MZI operation

The THz antenna coupled PPSs on the arm of the MZI introduce a phase delay Δ*φ*_THz_ proportional to the THz electric field (*E*_THz,g_). PPSs with opposite crystal orientations in the two branches of the MZI introduce, upon an incident field, opposite and thus additive phase shifts (push–pull configuration)^34^. The push–pull operation yields a total phase delay of Δ*φ* = 2Δ*φ*_THz_. The operating point of the MZI is chosen such that, after interference of the probe pulses at the output beam combiner, the output intensity is modulated linearly with the THz electric field (the so-called quadrature point of the MZI) (see Supplementary Note 2). A detailed analysis of the detector performance with respect to the MZI operation point is discussed in the Supplementary Note 3.

### Refractive index change

The change of the effective group index of the probe pulse in each PPS^35^ is ${\mathrm{\Delta }}n_{{\mathrm{eff}}} = \frac{1}{2}n_{{\mathrm{mat}}}^2r_{33}E_{{\mathrm{THz}},{\mathrm{g}}}n_{\mathrm{g}}\Gamma _{\mathrm{c}}$, where *n*_mat_ = 1.77 is the NLO refractive index at optical frequencies, *r*_33_ ≈ 120 pm V^−1^ is the nonlinear coefficient, *E*_THz,g_ is the THz electric field in the plasmonic slot, and *n*_g_Γ_c_ is the product of the group effective refractive index and the field energy confinement factor (see Supplementary Note 4).

### Electro-optic sampling

An electro-optic sampling setup as reported in ref. ^26^ has been modified to facilitate the fiber coupling of the probing pulses to and from the chip. Both the sampling probe pulses and the THz pulses are generated by a fiber-coupled IR fs laser at a 90 MHz rate. The large area PCA emitter is biased with a voltage of *V*_pp_ = 12 V. The time-domain setup is not purged and the THz pulses are then transmitted through free space for a length of ~50 cm before they reach the detector. The THz signal was focused on the chip by means of an off-axis parabolic mirror with a focal length of 76.2 mm. The focal length is chosen to allow enough space for the fiber coupler and chip mount. The PCA is pumped by 150 fs long pump pulses centered around 780 nm. All measurements except the linearity measurements were recorded at a pump power of 76 mW. By sweeping the delay between the IR probe and the THz pulse, a time-resolved THz field amplitude can be reconstructed from the different time samples. The time traces for the 1.2 and 2.4 THz detectors were measured with an integration time of 20 s per point at an optical probe power of 63 nW (corresponding to 0.7 fJ pulse energy). The recorded time traces were about 15 ps long with time steps of 48 fs. The length of the time trace gives a spectral resolution of 62 GHz. Nevertheless, some absorption lines (1.11, 1.36, 1.6, 2.1, and 2.35 THz) can be identified from the measured spectrum with the 2.4 THz detector and the 1 mm long ZnTe crystal (see Supplementary Note 6).

The reference measurements with ZnTe nonlinear crystals with a length of 200 μm and 1 mm were performed in a free-space optical setup including a quarter wave plate, a Wollaston prism and a balanced detector. An optical probe with a wavelength of 780 nm was used to maximize phase matching. The optical probe power on the detector was 900 μW and 1.8 mW, respectively. Note that for a 200 μm long ZnTe crystal phase matching is avoided, and we can assume this to be equivalent to the input pulse itself. The measured spectrum with the 1 mm and 200 μm long ZnTe crystals are shown in the Supplementary Note 6.

The linearity of our devices was measured by sweeping the optical pump power launched on the PCA. The THz field amplitude depends linearly on the pump power of the PCA.

### Potential of the concept to increase the dynamic range

The dynamical range is currently 65 dB. In this implementation it is limited by the optical power level at the output of the chip. However, this can be improved as follows. First, optical losses were rather high in the current chip generation (11.5 dB per grating coupler and 8 dB plasmonic losses). Using state-of-the-art silicon foundry coupling schemes would reduce the out-coupling losses by 10 dB^44^. Also, plasmonic losses of 3 dB for a 5 μm long device is possible^45^. Second, higher optical powers would be a solution to further increase the dynamical range. Yet, in the current implementation we did not increase the optical probe power in order to stay below the nonlinear threshold of the silicon waveguide (e.g. two-photon absorption) and the damage threshold of the nonlinear material. However, it should be emphasized that these are not fundamental limits. The first can be overcome by resorting to a waveguide material with a larger bandgap (e.g. silicon nitride) and wider cross sections. The latter threshold can be overcome by switching to more recent nonlinear materials. New organic materials have been shown to work under much higher temperatures^46,47^. Also, recent barium titanate (BTO) plasmonic modulators have shown great potential for high-power applications with demonstrated high-temperature stability beyond 250 °C^48^. With the improvements discussed above, 15 dB from reduced optical losses and 15 dB from higher optical input powers would increase the output power by up to 30 dB. This should increase the dynamical range from 65 to 95 dB, for shot noise limited detection (see Supplementary Note 8). Finally, one could use a 2 × 2 coupler at the output of the integrated MZI detector to allow for balanced detection. This would further improve the sensitivity of the detector.

## Supplementary information

Supplementary Information

## Data Availability

Detailed geometry of the detector is given in Supplementary Fig. 1, and all the dimensions are provided in Supplementary Tables 1 and 2. The simulated field enhancement of the detectors is given in Supplementary Fig. 2. A detailed MZI transfer function with indicated operation points is depicted in Supplementary Fig. 3. The MZI tuning structure with measured data is shown in Supplementary Fig. 4. The detector efficiency as a function of the MZI operation point is discussed in Supplementary Fig. 5. The optical mode and THz field confinement in the plasmonic waveguide is shown in Supplementary Fig. 6. The retrieved time-domain input signal amplitude profiles for the 1.2 and 2.4 THz detectors are shown in Supplementary Fig. 7. The spectral responses of the 1 mm and 200 μm long ZnTe crystals are given in Supplementary Fig. 8. The electro-optic sampling characteristics of different platforms are summarized in Supplementary Table 3. The dynamic range as a function of the probe power and modulation efficiency is discussed in Supplementary Fig. 9. The data that support the findings of this study are available from the corresponding authors upon reasonable request.
