# Supplementary material for: Compact and ultra-efficient broadband plasmonic terahertz field detector
Source: Nat Commun. 2019 Dec 5;10:5550. doi: 10.1038/s41467-019-13490-x (PMC6895079; doi:10.1038/s41467-019-13490-x)
Supplement: Supplementary file 1 — Supplementary Information [file 41467_2019_13490_MOESM1_ESM.pdf]

Supplementary information for

Compact and ultra-efficient broadband plasmonic terahertz field detector

Salamin et al.

Supplementary Note 1: THz antenna design and frequency response.

The THz detector comprises of an antenna and a plasmonic slot waveguide filled with a nonlinear material. The collected energy by this antenna can be optimally concentrated into the nonlinear material for maximum detection efficiency. An expression describing the field enhancement (FE), defined as the ratio between the electric field in the plasmonic slot ($E_{\mathrm{THz,g}}$) and the incident electric field ($E_{\mathrm{THz,i}}$), as a function of the device parameters was previously derived[1]

$$\mathrm{FE} = \frac{E_{\mathrm{THz,g}}}{E_{\mathrm{THZ,i}}} = \frac{\sqrt{Z_{\mathrm{d}}}}{w_{\mathrm{slot}}} \cdot \sqrt{\frac{\lambda_{\mathrm{THz}}^2 G_{\mathrm{R}}}{4\pi Z_0}} \tag{1}$$

where Z_{d} is the device's impedance, w_{slot} is the plasmonic slot width, λ_{THz} is the THz wavelength, G_{R} is the antenna gain and Z_0 is the free-space impedance. Clearly, the slot width has a strong impact on the efficiency. The nanoscale plasmonic slot can therefore enable optimal field enhancement. In addition, maximum voltage drop (electric field) in the nonlinear material is achieved for a device with a high impedance. This is achieved by exploiting the full-wave resonance of the antenna[2]. Finally, strong resonance, i.e. high impedance, can be achieved with magnetic resonators such as the four-leaf clover antenna. In such a structure, the inductive part comes mainly from the split ring features, and therefore, wider antenna arms can be used to reduce ohmic resistance and increase the antenna quality (Q) factor. This however comes with a bandwidth-efficiency trade-off. In other words, the stronger the resonance the narrower the bandwidth. A broadband antenna response can still be achieved by combining multi-resonance structures.

Supplementary Figure 1a shows a microscope image of the detector, including the electrical contacts used for electric field poling. The total dimensions (L_{ant} x W_{D}) of the detectors are 649 x 124 μm^2 and 318 x 89 μm^2 for the 1.2 THz and 2.4 THz detector, respectively. Supplementary Figure 1b shows a schematic view of the antenna structure with crucial parameters. All parameter values for both antennas are summarised in Supplementary Table 1. All parameters are given in micrometres (μm).

Supplementary Table 1 Antenna dimensions in micrometres.

	D_x	D_y	G_x	G_y	w	L_1	L_c	L_i	w_c	N_c	h_{Au}	L_{ant}	W_{D}
**1.2 THz**	29	32	11	2.2	3.7	40	30	30	3.7	5	0.15	649	124
**2.4 THz**	10	15.5	5.8	2.3	1	19	15	15	1	5	0.15	318	89

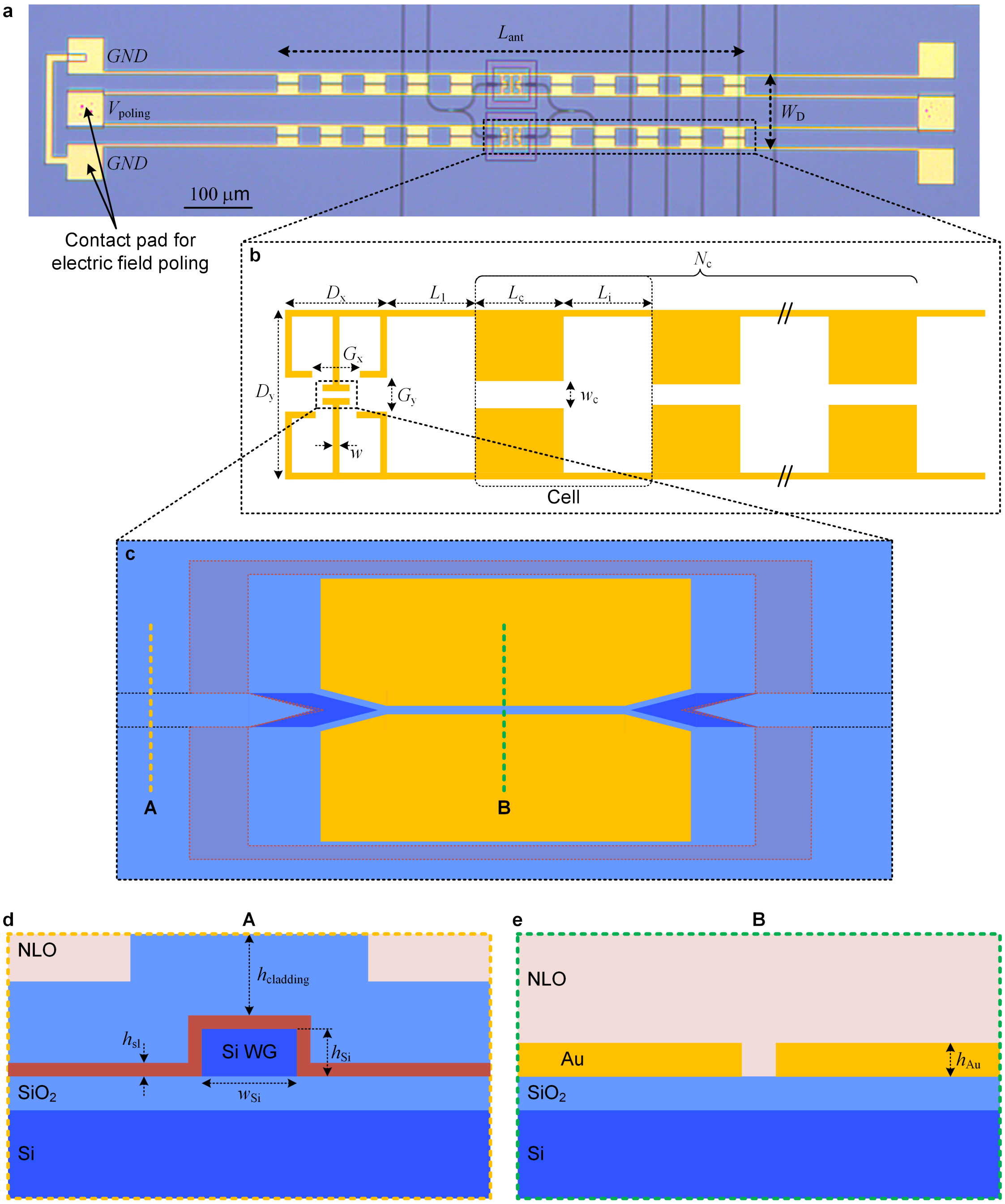

Supplementary Figure 1 Device geometry. **a** Microscope image of the fabricated device. **b** Schematic view of the antenna. Note that the antenna is symmetric but only one side of the PBG structure is shown. **c** Top schematic view of the plasmonic slot section. The shaded red area indicates where the silicon dioxide cladding is locally removed. The inner boundary is where the etch stop layer is additionally removed. **d** Cross-sectional view (**A**) of the Si waveguide. The dimensions of the waveguide are w_{Si}=450 nm and h_{Si}=220 nm, and the SiO_2 cladding $h_{cladding}$ is 700 nm height. **e** Cross-sectional view (**B**) of the plasmonic waveguide. Height of the metal is 150 nm.

A detailed top-view of the active section (plasmonic waveguide) including the silicon access waveguide and photonic-plasmonic coupler is shown in Supplementary Figure 1c. The SiO_2 cladding covering the whole chip is locally removed around the active section of the device, see

depicted area in Supplementary Figure 1c. A 100 nm alumina layer before the cladding deposition is used as the etch stop layer when opening the SiO_2 cladding. The etch stop layer is additionally removed (inner boundary of the shaded area in Fig. S1c). The cross-section view of the silicon waveguide (**A**) and the plasmonic waveguide (**B**) are respectively shown in Supplementary Figure 1d and e, respectively. The silicon waveguide dimensions are given in Supplementary Table 2. All parameters are given in micrometres (μm).

Supplementary Table 2 Silicon waveguide dimensions in micrometres.

w_{Si}	h_{Si}	$h_{cladding}$	h_{sl}
0.45	0.22	0.7	0.1

The detector was optimized for the reception of THz signals by combining the antenna with phase shifters integrated into a MZI. This allows for directly measuring the electric field amplitude and phase in absolute units (V m^{-1}) for frequencies up to several THz. It should also be noted that the THz electric field received by both antennas (in both MZI arms) has the same electric field polarity. So for the detector, as opposed to traditional MZI modulators[3], the nonlinear material in the two phase shifter arms should come with an opposite orientation in order to achieve push-pull operation[3]. This is achieved by poling the device asymmetrically between the two antennas, see Supplementary Figure 1a.

The broad frequency response of the antenna can be decomposed into two parts, i.e. high- and low-frequency. The simulated field enhancement of the low- (dashed curves) and high-frequency (dotted curves) antenna parts of the 1.2 THz and 2.4 THz detector are shown in Supplementary Figure 2a and b, respectively. The part of the antenna providing the high-frequency response is the four-leaf clover (4LC) resonator at the centre of the structure (Fig. 1c in the manuscript). The 4LC resonator is designed for a strong field enhancement within a reasonably broad spectral window. The geometry is therefore ideally suited for the high frequency response of the antenna, considering that the THz source is weakest at highest frequency. For the low frequency part of the spectrum, photonic-band gap (PBG) lines acting as a Bragg mirror, provides several resonances in the frequency range of 0.1-0.7 THz. The photonic bandgap structure is optimized such as to minimize the influence on the high-frequency resonance provided by the 4LC structure, and simultaneously provide a broad field enhancement at low frequencies. The simulated overall field

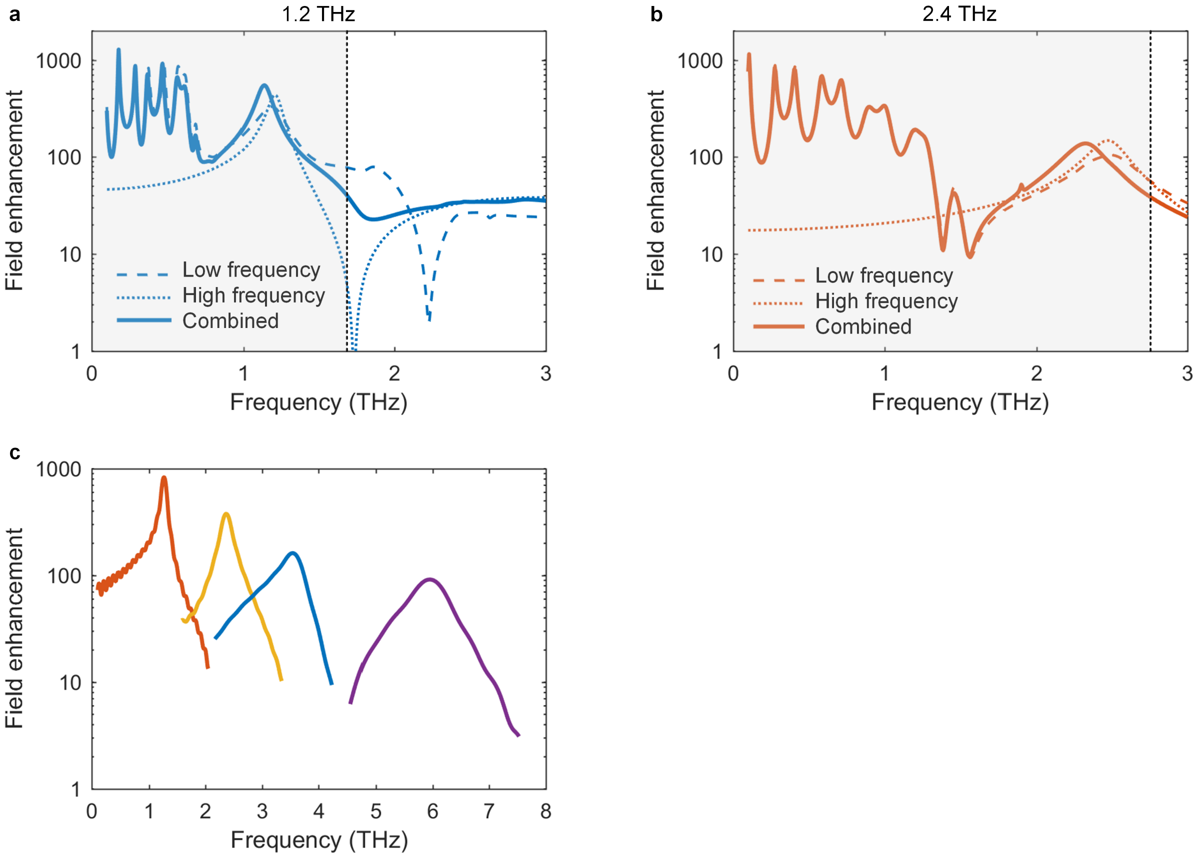

Supplementary Figure 2 Frequency response of the terahertz antenna. **a** and **b** Individual field enhancement contributions of the low frequency (dashed) and high frequency (dotted) part of the antenna for the 1.2 THz (blue lines) and 2.4 THz (red lines) detectors, respectively. In addition, the combined field enhancement of the 1.2 THz and 2.4 THz antenna is given for both structures (solid lines). The shaded area indicate the expected measurable signal. **c** Field enhancement for four-leaf clover antenna structure for different spectral windows.

enhancement (solid lines) provided by the 1.2 THz and 2.4 THz detectors are also shown in Supplementary Figure 2a and b, respectively. By controlling the 4LC resonance and PBG geometry, the overall frequency response of the device can be tuned. The scheme offers the opportunity to optimize the RLC resonance to a wide variety of THz fields. For instance, a single 4LC resonator could be optimized with a centre frequency of 6 THz, see purple curve in Supplementary Figure 2c. However, such an antenna offers a limited passband which depends on the Q factor of the resonator. The higher the Q factor the higher the sensitivity of the detector, but the lower the bandwidth. However, using an integrated circuit allows for parallelization. One could align multiple detectors in parallel, each optimized for a different spectral window, as shown in Supplementary Figure 2c. It would also be possible to use different kinds of antennas with, for example, a more broadband response (bowtie antenna) or polarization in-sensitive (log-spiral antenna). It should be noted that, although the simulations help to understand the device operation and give a rough estimate of the expected frequency range, it is only part of the story.

Supplementary Note 2: Operation principle of the Mach-Zehnder interferometer.

The working principle of the Mach-Zehnder Interferometer (MZI) is discussed in this section with respect to its sensitivity to an externally applied THz field of amplitude $E_{\mathrm{THz,g}}$. For this purpose, we assume an input probe intensity I_{in} (which is proportional to the square of an electric field E_{in}) which is fed to the MZI. The probe signal is first split and further propagates through the two branches of the interferometer. Subsequently we will show, that the path imbalance between the two branches of the interferometer at zero applied THz field greatly determines the sensitivity of the interferometric detector. The path difference can be given by the phase offset $\varphi_{\mathrm{offset}} = \frac{2\pi}{\lambda_{\mathrm{p}}} n_{\mathrm{diel}} \Delta l$, with λ_{p} the center wavelength of the probe pulse, n_{diel} the mode refractive index of the

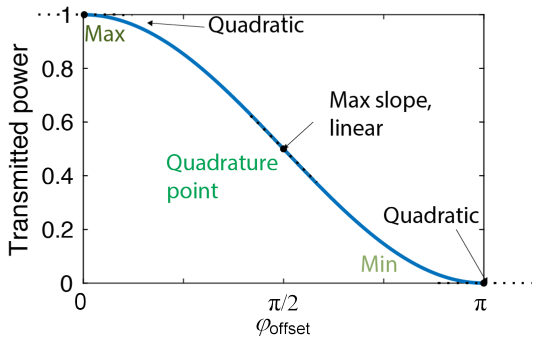

Supplementary Figure 3 Mach-Zehnder interferometer transfer function. The quadrature point with the linear regions is shown. The quadratic dependence point (max and min) are also shown.

probe pulse in the dielectric waveguide and Δl the geometric path difference between the two branches. We further assume L_{slot} to be the length of the plasmonic slot, α the attenuation coefficient of the probe intensity in the plasmonic slot and $\Delta\varphi_{\mathrm{THz}} = \frac{2\pi}{\lambda_{\mathrm{p}}} \Delta n_{\mathrm{eff}} L_{\mathrm{slot}} = \Delta\beta_{\mathrm{eff}} L_{\mathrm{slot}}$ the phase delay introduced by the in-gap THz electric field $E_{\mathrm{THz,g}}$. Δn_{eff} can be modulated upon applying an electric field $E_{\mathrm{THz,g}}$. Also, it should be noted, that in a MZI that is operated in push-pull operation mode one will induce a phase shift of $\Delta\varphi_{\mathrm{THz}}$ in one arm and a phase shift of $-\Delta\varphi_{\mathrm{THz}}$ in the other arm. The total phase shift thus is $2\Delta\varphi_{\mathrm{THz}}$. We assume $\Delta\varphi_{\mathrm{THz}} \ll \frac{\pi}{2}$ to be very small. The electric field and intensity of the probe after the interferometer then is

$$E_{\mathrm{out}} = E_{\mathrm{in}}\, e^{-\frac{\alpha}{2} L_{\mathrm{slot}}} \cos\left(\frac{\varphi_{\mathrm{offset}}}{2} + \frac{2\,\Delta\varphi_{\mathrm{THz}}}{2}\right) \qquad (2)$$

$$I_{\mathrm{out}} = \frac{I_{\mathrm{in}}}{2}\, e^{-\alpha L_{\mathrm{slot}}} [1 + \cos(\varphi_{\mathrm{offset}} + 2\,\Delta\varphi_{\mathrm{THz}})] \qquad (3)$$

Clearly, since

$$\frac{\partial I_{\text{out}}}{\partial \Delta\varphi_{\text{THz}}} = -I_{\text{in}}\, e^{-\alpha L_{\text{slot}}} \sin(\varphi_{\text{offset}} + 2\,\Delta\varphi_{\text{THz}}) \tag{4}$$

the sensitivity of the output intensity to an incident THz field is highest for $\sin(\varphi_{\text{offset}} + 2\,\Delta\varphi_{\text{THz}}) = 1$, that is for $\varphi_{\text{offset}} = \pi/2$ (quadrature point).

If the MZI is operated near the maximum transmission point, then $\varphi_{\text{offset}} \approx 0$ and

$$I_{\text{out}} = \frac{I_{\text{in}}}{2}\, e^{-\alpha L_{\text{slot}}}(2 - \frac{\varphi_{\text{offset}}^2}{2} - 2\,\Delta\varphi_{\text{THz}}^2 - 2\varphi_{\text{offset}}\,\Delta\varphi_{\text{THz}}) \tag{5}$$

$$\Delta I = I_{\text{out}} - I_{\text{out}}|_{\Delta\varphi_{\text{THz}}=0} = I_{\text{in}} e^{-\alpha L_{\text{slot}}}(\,\Delta\varphi_{\text{THz}}^2 + \varphi_{\text{offset}}\,\Delta\varphi_{\text{THz}}). \tag{6}$$

Hence, the intensity modulation contains a quadratic and a linear dependence on the THz electric field. Since typically we have, $\varphi_{\text{offset}} > \Delta\varphi_{\text{THz}}$, the linear term dominates. A similar behaviour occurs for $\varphi_{\text{offset}} \approx \pi$.

Conversely, if the MZI is operated near the quadrature point, then $\varphi_{\text{offset}} \approx \frac{\pi}{2}$ and

$$I_{\text{out}} = \frac{I_{\text{in}}}{2}\, e^{-\alpha L_{\text{slot}}}(1 + 2\,\Delta\varphi_{\text{THz}}) \tag{7}$$

$$\Delta I = I_{\text{out}} - I_{\text{out}}|_{\Delta\varphi_{\text{THz}}=0} = I_{\text{in}} e^{-\alpha L_{\text{slot}}}\,\Delta\varphi_{\text{THz}}. \tag{8}$$

In this point, the average output intensity is $\frac{I_{\text{in}}}{2}$ and the intensity modulation depends only linearly on the THz electric field.

Supplementary Note 3: MZI tuning with thermo-optic phase shifter.

In this section we discuss the performance of the demonstrated THz detector with respect to the proper choice of the operation point within the MZI. More precisely, there is a freedom to adjust a phase offset φ_{offset} on the two arms of the MZI by means of a thermoelectric heater and in this way to change the operation point of the transfer function of the MZI. The geometry of the detector is shown in Supplementary Figure 4c. Before the plasmonic phase shifters, the probe beams pass through a dielectric Si waveguide which is covered by an Ohmic resistor. Upon an applied current, the resistor dissipates heat, which in turn tunes the imbalance between the two arms of the interferometer. Upon tuning, the amplitude of the electro-optic signal and the total transmission through the interferometer is changed. Supplementary Figure 4a shows the normalized transmitted optical power as a function of the phase offset φ_{offset} between the two MZI arms, i.e. the transfer function.

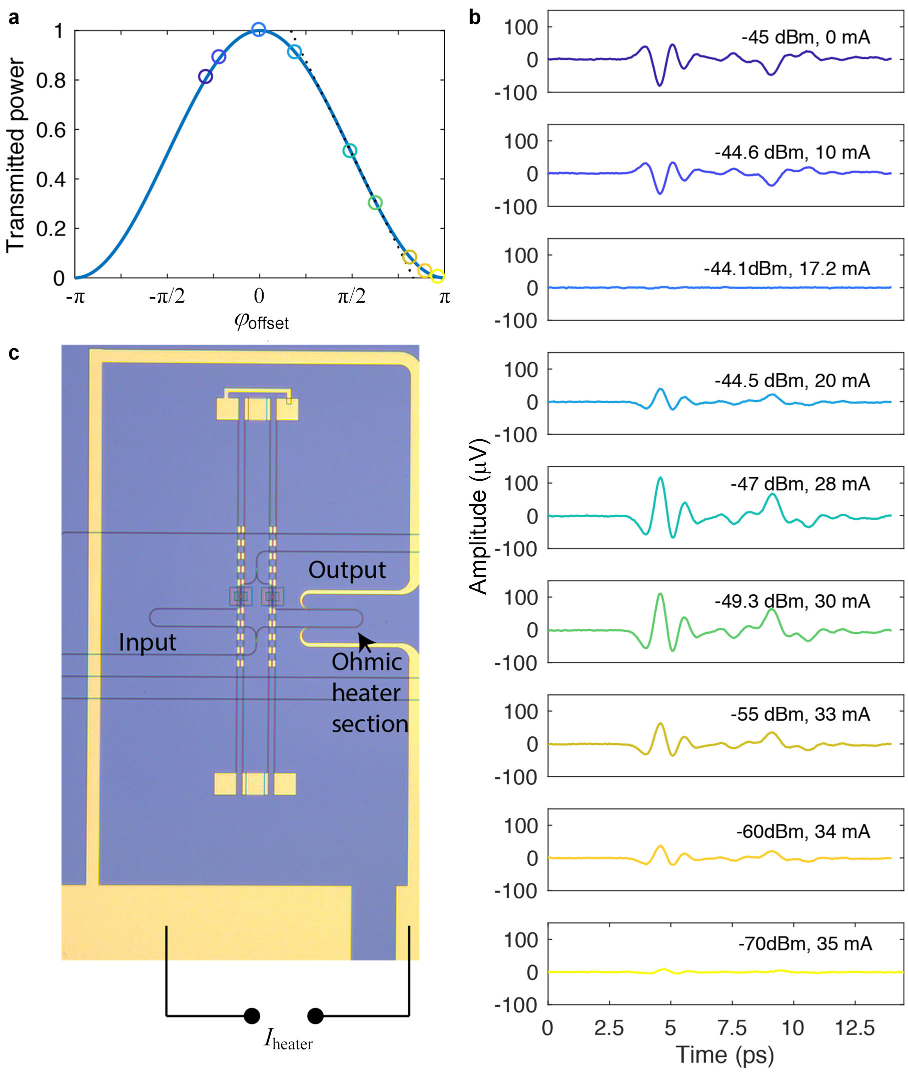

Supplementary Figure 4 On-chip interferometer tuning. **a** Measured transmitted power as a function of the phase offset between the MZI branches. **b** Measured time traces. The colours correspond to the measured transmitted power in **a**. **c** MZI THz detector structure with thermo-optic heater.

In Supplementary Figure 4b we show the detected time-traces for different phase offsets, with the corresponding phase offsets shown in Supplementary Figure 4a. We annotate in the legend also the measured output power and applied current to the thermo-optic heater to tune the MZI. When the interferometer has zero phase offset, i.e. $\varphi_{\text{offset}} = 0$, maximum optical power is transmitted, i.e. -44.1 dBm. It is worth noting that when the phase offset between both MZI arm changes from a negative offset ($\varphi_{\text{offset}} < 0$) to a positive offset ($\varphi_{\text{offset}} > 0$), the measured field amplitude changes sign accordingly.

Next, we discuss the THz detector performance with respect to its MZI operation point as summarised in Supplementary Figure 5a. For this purpose, we analyse the total peak-to-peak

modulation $\Delta I_{\mathrm{pp}} = \Delta I_{\max} - \Delta I_{\min}$ of the probe intensity (see left y-axis of Supplementary Figure 5a) as obtained from the measurements shown in Supplementary Figure 4b. $\Delta I_{\max}$ and $\Delta I_{\min}$ correspond to the maximal positive and negative THz field, respectively. In addition, we normalize ΔI_{pp} by the detected average intensity $\langle I \rangle$ to exclude the linear dependence of the electro-optic signal on the probing pulse intensity (eq. 1 in manuscript) and demonstrate the performance of the detector itself. We name this ratio the modulation efficiency η, i.e. $\eta = \Delta I_{\mathrm{pp}}/\langle I \rangle$ (see right y-axis of Supplementary Figure 5a).

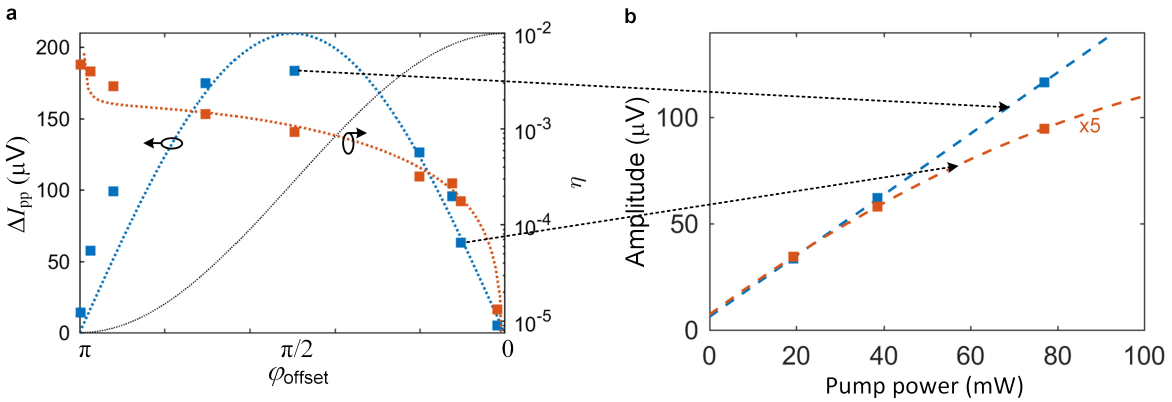

Supplementary Figure 5 Detector efficiency and linearity as a function of the MZI operation point. **a** Terahertz detector efficiency as a function of the MZI operation point. The black dotted curve shows the transmitted intensity as a function of the MZI operation point, i.e. the MZI transfer function, going from the dark state ($\varphi_{\mathrm{offset}} = \pi$ relative phase shift) to the bright state (0 relative phase shift). On the left y axis, we show the measured (blue squares) and expected (blue dotted line) absolute peak-peak (ΔI_{pp}) amplitude at a constant input probe intensity. The right y-axis plots the measured (red squares) and expected (red dotted line) modulation efficiency η. **b** The linearity of the electro-optic signal peak amplitude is investigated for the quadrature point where the response is linear and for the bright state where the response becomes quadratic.

In case the phase offset between the two MZI arms is $\varphi_{\mathrm{offset}} = \pi$, we are in a destructive operation mode – the so called Null-point operation point. Unfortunately, the intensity modulation around the Null point is then weak. Although the modulation efficiency η is maximal, not enough optical power reaches the photodiode to detect the THz field. Conversely, when the phase offset is $\varphi_{\mathrm{offset}} = 0$, maximum light reaches the photodiode. However, due to low modulation efficiency η, no THz field is detected as well. Furthermore, in the vicinity of the maximum transmission $\varphi_{\mathrm{offset}} = 0$, the response of the detector contains linear and quadratic terms (see equation in Supplementary Note 2), as shown in Supplementary Figure 5b. In other words, the detector becomes a photon detector. Finally, when the phase offset is set to $\varphi_{\mathrm{offset}} = \frac{\pi}{2}$, i.e. quadrature point of the MZI, the intensity modulation becomes largest and a maximal THz field amplitude

modulation is detected (see Supplementary Figure 5a). Also, the response of the detector is linear in field, as shown in Supplementary Figure 5b.

Supplementary Note 4: Electric field induced phase modulation.

Here we discuss the induced phase shift of the optical probe signal, i.e. phase modulation, by the applied THz electric field. In an electro-optic material, the phase shift is caused by the change of refractive index Δn_{mat} induced by the applied electric field. The accumulated phase shift over a distance L is then given by

$$\Delta\varphi = L \cdot \Delta\beta \tag{9}$$

where β is the propagation constant. For a small change in refractive index, the phase shift can be rewritten in terms of the group velocity $v_{\mathrm{g}} = \frac{\Delta\omega}{\Delta\beta}$,

$$\Delta\varphi \approx L \cdot \frac{\Delta\omega}{v_{\mathrm{g}}} = L \cdot \frac{n_{\mathrm{g}}}{c} \cdot \Delta\omega \tag{10}$$

where $n_{\mathrm{g}} = \frac{c}{v_{\mathrm{g}}}$ is the group index and c the speed of light. Note that $L \cdot \frac{n_{\mathrm{g}}}{c} = \frac{\mathrm{d}\varphi}{\mathrm{d}\omega} = \tau_{\mathrm{g}}$ is the group delay. It can be seen that the phase shift can be enhanced in low group velocity systems[4]. The group index is determined by the overlap integral of the energy stored in the field and the propagating Poynting vector[5]

$$n_{\mathrm{g}} = \frac{c}{v_{\mathrm{e}}} = c\,\frac{\iint \left(\varepsilon_0 \frac{d(\varepsilon(\omega)\omega)}{d\omega} EE^{+} - \mu_0 HH^{+}\right) dS}{\iint \Re(E \times H) \cdot \hat{\boldsymbol{z}} dS} \tag{11}$$

where v_{e} is the energy velocity[6], ε is the permittivity, $\Re$ the real part and $\hat{\boldsymbol{z}}$ is the unit vector in z-direction.

The shift in the optical propagation mode[4] due to a small Δn_{mat} is given by $\frac{\Delta\omega(\beta)}{\omega} = -\Gamma_{\mathrm{c}} \left(\frac{\Delta n_{\mathrm{mat}}}{n_{\mathrm{mat}}}\right)$, where Γ_{c} is the fraction of the total energy of the optical signal that contributes to the nonlinear interaction. In other words, it is the overlap integral between the energy of the optical component aligned to the nonlinear axis of the electro-optic material and the total optical energy[5]:

$$\Gamma_{\mathrm{c}} = \frac{\varepsilon_{\mathrm{r}} \iint_{\mathrm{gap}} |E_{\mathrm{x}}|^2 dS}{\iint \left(\varepsilon_0 \frac{d(\varepsilon(\omega)\omega)}{d\omega} EE^{+} - \mu_0 HH^{+}\right) dS}. \tag{12}$$

We can rewrite the induced phase shift

$$\Delta\varphi = L \cdot k_0 \cdot \Gamma_c \frac{\Delta n_{\text{mat}}}{n_{\text{mat}}} n_g. \tag{13}$$

where $k_0 = \frac{\omega}{c}$ is the wavenumber. The linear electro-optic effect introduces a change in the refractive index of the nonlinear material upon an applied electric field $E_{\text{THz,g}}$

$$\Delta n_{\text{mat}} = \frac{1}{2} n_{\text{mat}}^3 r_{33} E_{\text{THz,g}}, \tag{14}$$

Finally, the induced phase change can be written as

$$\Delta\varphi_{\text{THz}} = \frac{1}{2} n_{\text{mat}}^2 r_{33} E_{\text{THz,g}} L k_0 \Gamma_c n_g. \tag{15}$$

Supplementary Figure 6a shows the field profile for the optical probe signal (1560 nm) and the THz signal (2.4 THz) for a slot width of 100 nm computed with COMSOL and CST Microwave Studio, respectively. Both signals are well confined to the plasmonic gap with an almost perfect overlap. Supplementary Figure 6b plots the computed effective group index change and the induced phase shift, for a plasmonic slot width of 100 nm and length of 5 µm. An electro-optic coefficient of 120 pm/V is assumed.

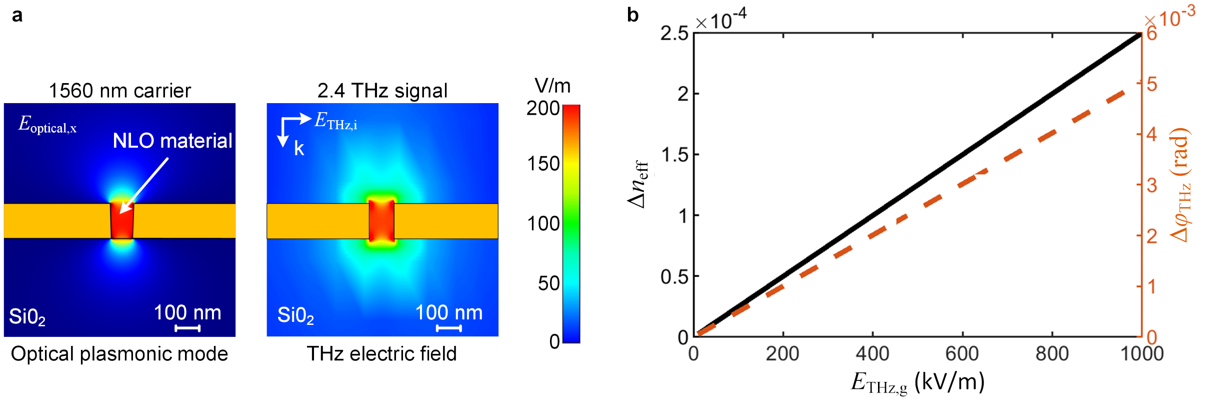

Supplementary Figure 6 Plasmonic phase shifter. **a** Optical and THz field distribution. **b** Computed change in effective group index (black curve) and induced phase shift (red curve) for a 2.4 THz device with a slot width of 100 nm and a length of 5 µm.

Supplementary Note 5: THz detector filter response.

Following the same procedure as described in the main manuscript, an approximated filter response $H'(\omega)$ for the 2.4 THz detector was obtained. In Supplementary Figure 7 we show the retrieved signals $s'(t)$ using the approximated filter response for both the 1.2 THz and 2.4 THz

detectors. It can be seen that both pulses show great similarities, indicative of a good approximation of the filter response.

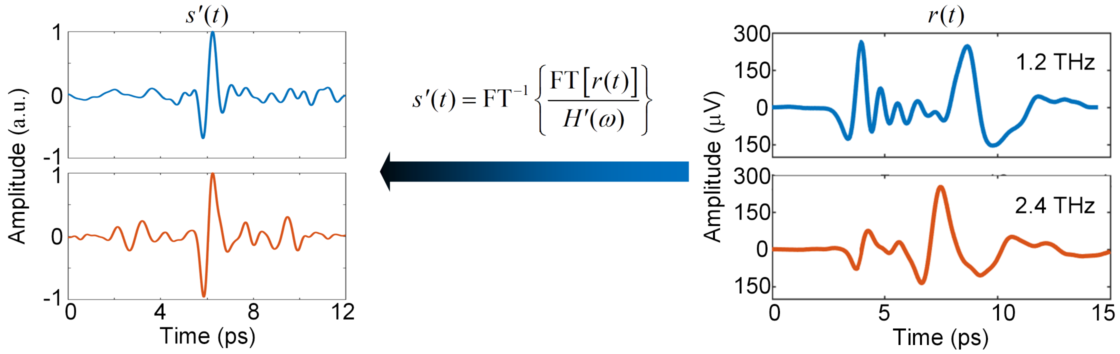

Supplementary Figure 7 Time domain response retrieved with the approximated filter response for the 1.2THz (blue curves) and 2.4THz (red curves) detectors.

Supplementary Note 6: Spectral response of ZnTe and water absorption lines.

Supplementary Figure 8 shows the measured spectral response with the 1 mm and 200 μm long ZnTe crystals, and the 2.4 THz MZI detector. It can be seen how the spectral response of the 2.4 THz detector compares nicely with the 1 mm long ZnTe crystal. Several water absorption lines are visible in the spectrums (see dashed black lines). The absorption line frequencies are indicated in THz unit[7].

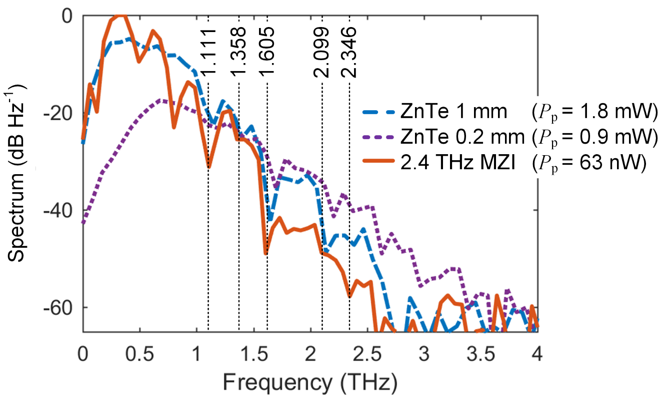

Supplementary Figure 8 Spectral response measured with the 1 mm and 0.2 mm long ZnTe crystals, and the 2.4 THz MZI detector. The probe power P_p used to measure the different spectrums are 1.8 mW, 0.9 mW and 63 nW, respectively. Visible water absorption lines are shown. The numbers indicate the absorption frequency in THz

The small frequency deviations in absorption lines are due to the low spectral resolution. The spectral response of the 200 μm long ZnTe exhibits a smaller dynamic range but with a larger bandwidth. This is because the coherence length doesn’t limit the detection bandwidth. Note that the ZnTe spectrums for the 1 mm and 200 μm long ZnTe crystal were taken with an optical probe

power of 1.8 mW and 900 μW, respectively. The 2.4 THz MZI detector was measured with an optical probe power in the photodiode of 63 nW.

Supplementary Note 7: Comparison among different technologies for THz electric field measurement.

We further compare different platforms that are commonly used for coherent detection of the electric field of terahertz (THz) waves with the purpose to evaluate their individual performance and potential for improvement. In that respect, we shall compare electro-optic detection in ZnTe crystal 1 mm, photoconductive detector at 1550 nm, large-area photoconductive detectors at 780 nm, detection in organics combined with a three-dimensional antenna and the organic-plasmonic on-chip detectors. We will resort to distinct scientific reports about the performance of the individual detectors and they will be used to benchmark the different technologies. This study is intended to give an overview over the existing technology and is not aspiring to provide an ultimate comparison as all experiments have been done in different laboratories and in different experimental configurations.

In the different platforms, the electric field of a terahertz wave is measured indirectly, by means of a femtosecond probe. For instance, in electro-optic detection schemes the THz electric field ultimately introduces a modulation of the intensity of the probe beam. Conversely, in photoconductive detection schemes the probe beam generates carriers in a semiconductor which are then accelerated by the THz wave to produce a photocurrent that is finally detected.

In the case of free-space electro-optic detection in ZnTe 110-cut, the intensity modulation is equal to:

$$\Delta I(\tau) = \frac{I_{\mathrm{p}} r_{41} n_{\mathrm{opt}}^3 l_{\mathrm{int}} \omega E_{\mathrm{THz}}(\tau)}{c_0} \tag{16}$$

with I_{p} the total probe intensity travelling through the detection crystal, ω the angular frequency of the probe, n_{opt} the refractive index at the probe frequency, l_{int} the interaction length between the two beams, and r_{41} the electro-optic coefficient of ZnTe. Typically, $I_{\mathrm{p}} = 2$ mW, $n_{\mathrm{opt}} = 2.8$, $r_{41} = 3.9\ \mathrm{pm\ V^{-1}}$, $l_{\mathrm{int}} = 1$ mm and $\lambda_0 = 800$ nm. Therefore,

$$\frac{\Delta I(\tau)}{I_{\mathrm{p}}} = 6.67 \cdot 10^{-7} \frac{\mathrm{m}}{\mathrm{V}} E_{\mathrm{THz}}(\tau). \tag{17}$$

In the case of an electro-optic detection scheme inside the three-dimensional antenna with the probe coupled from free-space:

$$\Delta I(\tau) = \frac{I_{\mathrm{p}} r_{33} n_{\mathrm{opt}}^{3} l_{\mathrm{int}} \omega E_{\mathrm{THz}}(\tau) \Gamma \cdot \mathrm{FE}}{c_0} \tag{18}$$

with r_{33} the electro-optic coefficient of the nonlinear organic material, Γ the mode overlap and FE the field enhancement provided by the antenna. Typically, $I_{\mathrm{p}} = 0.074$ mW, $n_{\mathrm{opt}} = 1.76$, $r_{33} = 120$ pm V^{-1}, $l_{\mathrm{int}} = 0.004$ mm, FE $= 100$ and $\lambda_0 = 1550$ nm. In this case, Γ is very small as the mode matching of the free-space probe to the antenna gap is highly limited by diffraction and misalignment. If we assume $\Gamma = 0.5$, then

$$\frac{\Delta I(\tau)}{I_{\mathrm{p}}} = 5.3 \cdot 10^{-7} \frac{\mathrm{m}}{\mathrm{V}} E_{\mathrm{THz}}(\tau). \tag{19}$$

In the case of the integrated electro-optic THz detector with the MZI configuration featuring a hybrid organic-plasmonic phase modulators one finds:

$$\Delta I(\tau) = \frac{I_{\mathrm{p}} r_{33} n_{\mathrm{opt}}^{2} l_{\mathrm{int}} \omega E_{\mathrm{THz}}(\tau) \Gamma_{\mathrm{c}} n_{\mathrm{g}} \cdot \mathrm{FE}}{c_0} \tag{20}$$

with $\Gamma_{\mathrm{c}} n_{\mathrm{g}}$ the field energy interaction factor. Typically, $I_{\mathrm{p}} = 10^{-4}$ mW, $n_{\mathrm{opt}} = 1.77$, $r_{33} = 120$ pm V^{-1}, $\Gamma_{\mathrm{c}} n_{\mathrm{g}} = 1.6$, $l_{int} = 0.004$ mm, FE $= 1000$ and $\lambda_0 = 1550$ nm. Therefore,

$$\frac{\Delta I(\tau)}{I_{\mathrm{p}}} = 7.62 \cdot 10^{-6} \frac{\mathrm{m}}{\mathrm{V}} E_{\mathrm{THz}}(\tau) \tag{21}$$

We find therefore that the use of a MZI detector featuring hybrid organic-plasmonic phase shifters of a width of 75 nm yields an at least ten times higher electro-optic signal than all other detection schemes at equal probe powers. For shot-noise limited detection, the SNR of the MZI detector is thus at least a factor of 10 higher than the other detection schemes at equal probe powers.

In the case of a photoconductive detector, we find that the photocurrent driven by the terahertz electric field at the electrodes is equal to[8]:

$$I(\tau) = \frac{\mu_0 \mathrm{e} P_{\mathrm{G}} T_{12} \lambda}{h c_0 \Sigma} \int_{-\infty}^{\infty} E_{\mathrm{THz}}(t)\, \Phi(t-\tau) dt \tag{22}$$

where μ_0 is the electron mobility in the semiconductor and e the elementary charge. $\Phi(t-\tau)$ describes the temporal (and thus frequency) response of the semiconductor and is determinded by the carrier lifetime. T_{12} is the Fresnel transmission coefficient for light from a laser beam of center

wavelength λ and waist of standard deviation Σ. P_G is the average power entering the semiconductor, h is the Planck's constant and c_0 is the speed of light in vacuum.

We summarize different characteristics of the different platforms in Supplementary Table 3. For near-field (NF) measurement possibility but with the probe still propagating through an analyte is marked with yes*.

Supplementary Table 3 Electro-optic sampling characteristics of different platforms.

Detection	η/E_{THz} [m V^{-1}]	l_{int}	Confinement	$E_{THz,i}$	P_{pump}	P_{probe}	FIBER	NF	DR [dB]
**This work**	$7.62 \cdot 10^{-6}$	5 µm	5 × 0.075 µm²	n.a.	76 mW	63 nW	yes	yes	65 @ 20 s
**ZnTe-780 nm**	$6.67 \cdot 10^{-7}$	1 mm	π(1 mm)²	n.a.	35 mW	1.8 mW	no	no	80 @ 1 s
**3D Ant-1550 nm**[9]	$5.3 \cdot 10^{-7}$	4 µm	5 × 2.2 µm²	n.a.	76 mW	74 µW	no	yes*	40 @ 5 s
**PCA-1550 nm**[10]	n.a.	n.a.	n.a.	75 µW	20 mW	10 mW	yes	yes*	100 @ 70 ns
**PCA-780 nm**[11]	n.a.	n.a.	n.a.	n.a.	n.a.	170 mW	no	yes*	107 @ 7.5 s

Supplementary Note 8: Influence of probe power and modulation efficiency on dynamic range.

Supplementary Figure 9 shows the dynamic range (DR) as a function of the probe power and the modulation efficiency. The measured DR for the 2.4 THz antenna and the 1 mm long ZnTe crystal are indicated in white for similar measurement specifications. The integrated THz detector has a strong efficiency but a limited probe power. On the contrary, the ZnTe crystal has a low modulation efficiency but a high optical power. Assuming the improvement in optical power as discussed in the method section of the manuscript, an at least equal dynamic range (red point) would be possible for the integrated detector of only 5 µm in length.

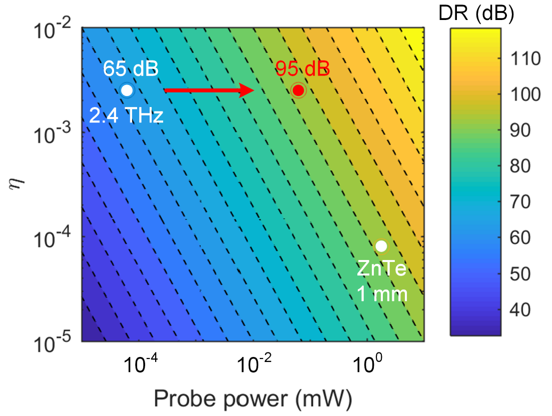

Supplementary Figure 9 Dynamic range as a function of the probe power and modulation efficiency.

Supplementary References

1 Salamin, Y. et al. Direct Conversion of Free Space Millimeter Waves to Optical Domain by Plasmonic Modulator Antenna. *Nano Lett.* **15**, 8342-8346 (2015).

2 Woo, I., Nguyen, T. K., Han, H., Lim, H. & Park, I. Four-leaf-clover-shaped antenna for a THz photomixer. *Opt. Express* **18**, 18532-18542 (2010).

3 Heni, W. et al. 108 Gbit/s Plasmonic Mach-Zehnder Modulator with >70 GHz Electrical Bandwidth. *J. Lightw. Technol.* **34**, 393-400 (2016).

4 Soljačić, M. et al. Photonic-crystal slow-light enhancement of nonlinear phase sensitivity. *J. Opt. Soc. Am. B* **19**, 2052-2059 (2002).

5 Haffner, C. et al. All-plasmonic Mach–Zehnder modulator enabling optical high-speed communication at the microscale. *Nat. Photon.* **9**, 525-528 (2015).

6 Chen, P. Y. et al. Group velocity in lossy periodic structured media. *Physical Review A* **82**, 053825 (2010).

7 Allman, R. E. & Foltynowicz, R. J. Terahertz time-domain spectroscopy of atmospheric water vapor from 0.4 to 2.7 THz. Report No. SAND2005-5709, (Sandia National Laboratories, 2005).

8 Castro-Camus, E. et al. Photoconductive response correction for detectors of terahertz radiation. *Journal of Applied Physics* **104**, 053113 (2008).

9 Benea-Chelmus, I.-C. et al. Three-Dimensional Phase Modulator at Telecom Wavelength Acting as a Terahertz Detector with an Electro-Optic Bandwidth of 1.25 Terahertz. *ACS Photon.* **5**, 1398-1403 (2018).

10 Globisch, B. et al. Iron doped InGaAs: Competitive THz emitters and detectors fabricated from the same photoconductor. *J. Appl. Phys.* **121**, 053102 (2017).

11 Yardimci, N. T. & Jarrahi, M. High Sensitivity Terahertz Detection through Large-Area Plasmonic Nano-Antenna Arrays. *Sci. Rep.* **7**, 42667 (2017).
